# Analyzing the relationship between pricing strategy and customer retention in hotels: A study in Albania

**DOI:** 10.12688/f1000research.132723.1

**Published:** 2023-06-16

**Authors:** Brunela Trebicka, AZETA TARTARAJ, Ariola Harizi

**Affiliations:** 1 Department of Applied Statistics and Informatics, Faculty of Business, Universiteti Aleksander Moisiu Durres, Durrës, Durrës County, Albania; 2 Department of Marketing, Faculty of Business, Universiteti Aleksander Moisiu Durres, Durrës, Durrës County, Albania

**Keywords:** Economy Pricing; pricing strategies; Customer retention; Hospitality industry; Relationship marketing

## Abstract

**Background:** In the consumer-centric global economy of the 21st century, customer retention is a vital concept in the hospitality industry for building and sustaining long-term relationships. Recognizing the challenges of acquiring new customers, the industry acknowledges the significance of retaining existing ones. To achieve their goals, hospitality businesses need a comprehensive strategy that extends beyond price targets, emphasizing the development of effective pricing strategies from the outset.

**Methods:** Data collection took place between June 2022 and January 2023, involving a random sample of seven international hotels located in Tirana, Durres, and Vlora. Quantitative data was collected through surveys utilizing Likert-scale questions. Statistical analysis, including crosstab tests, was employed to explore the relationship between economy pricing strategies and customer retention.

**Results**: The study encompassed 572 participants representing diverse demographic characteristics. Analysis revealed a statistically significant positive relationship between economy pricing strategies and customer retention in international hotels in Albania. These findings underscore the importance of implementing effective pricing strategies to enhance customer loyalty and guide the development of improved strategies within the hotel industry.

**Conclusions:** This study provides empirical evidence of a significant positive relationship between economy pricing strategies and customer retention in international hotels in Albania. Effective pricing strategies play a crucial role in fostering customer loyalty. However, the study's limitations, primarily its focus on specific hotels in Albania, call for further research to validate the generalizability of these findings. The insights gained from this study inform policymakers and industry stakeholders in formulating strategies to enhance customer retention within the hospitality sector.

## Introduction

Over the past decade, international hotels have undergone significant development, making it challenging for them to gain a competitive advantage in the highly competitive market environment. As a result, hotels have to adopt new strategies to achieve their long-term goals. One of the most significant changes has occurred in international hotel pricing, with the implementation of various pricing strategies. Pricing strategy is critical for the success of international hotels. The price must be competitive, accurate, and consistent ( Gao, Wang, and Fang, 2020).

An effective pricing strategy can improve business performance and lead to positive outcomes such as customer satisfaction, loyalty, and customer retention, especially for business entities ( Zakaria, Abdullah, and Ali, 2019). A pricing strategy is an essential part of creating a sustainable competitive advantage that helps businesses build customer loyalty and increase satisfaction. An effective pricing strategy gives a business an edge over competitors by delivering more value to customers at lower cost or higher price, where additional benefits and services are added. When a business spends less than its competitors, it can use lower prices to attract customers. Therefore, pricing is critical to business leadership. Moreover, companies use price reductions as a strategy to increase or maintain their market share. Customers often use price as an indicator of product quality, especially when they have limited information to make a judgment ( Rizov, 2019).

### Research problem

There are many studies on pricing strategies and customer retention in the hospitality industry, but little attention has been given to the impact of economy pricing strategies on hotel customer retention, especially in Albania. Therefore, this study aims to investigate the impact of economy pricing strategies on customer retention in selected international hotels in Albania.

The research aims to assess the level of use of economy pricing strategies and their impact on customer retention in the international hotel industry in Albania, with the aim of improving the development of hotel services in a competitive market environment.

This study will provide valuable insights into the effectiveness of cost-effective pricing strategies that comparable hotels can use to retain guests. The results will help hoteliers evaluate their current pricing strategies and make any necessary adjustments to improve guest retention.

Additionally, this research will help business leaders identify effective retention techniques and areas for improving customer retention policies.

### Research objectives

The main research objectives of this study are:

1. Investigate the relationship between economy pricing strategy and customer retention of international hotels in Albania.
2. Identify the most effective economy pricing strategy to achieve and increase customer retention of international hotels in Albania.

### Research questions

The following research questions will guide this study:

Research question one (RQ1): Is there a relationship between economic pricing strategies and customer retention in international hotels in Albania?

Research question two (RQ2): What is the impact of economy pricing strategies on customer retention in selected international hotels in Albania?

### Conceptual framework

**Figure 1.**
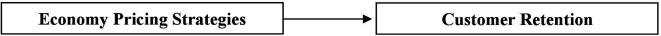
The relationship between pricing strategies and customer retention.

This study aims to investigate the relationship between economy pricing strategies and customer retention rates in selected international hotels in Albania. Economy pricing strategy is considered as the independent variable, while customer retention is the dependent variable. The conceptual framework in the Figure 1, below illustrates the relationship between the two variables:

Based on the relationship between the independent and dependent variables, the research hypothesis are as follow:

Null hypothesis (H _0_): There is no significant relationship between economy pricing strategies and customer retention in international hotels in Albania.

Hypothesis one (H _1_): There is a significant positive relationship between economy pricing strategies and customer retention in international hotels in Albania.

### Literature review

The importance of pricing is an important part of the marketing mix that generates revenue for a company ( Asamoah and Chovancová, 2011). It is a flexible variable that can be adjusted based on market demand ( Zhang *et al.,* 2017) states that price determines the amount buyers are willing to pay for a product or service based on their decision. Price is one of the four P’s of the marketing mix that consumers consider before making a purchase decision ( Kotler *et al.*, 2022a). When determining the pricing strategy, it is essential to consider three critical factors, including the perception of the product’s value by buyers, the number of buyers in the market, and their price sensitivity ( Azad and Subrahmanyam, 2022).

Furthermore, understanding how buyers will respond to prices requires research and analysis. Price elasticity, or the sensitivity of buyers to price changes, impacts the demand for products. The degree of change in demand in response to a change in price is known as price elasticity. According to Agwu (2018), when buyers are highly sensitive to price changes, they buy more of a product at lower prices and less of it when the price is higher. This indicates that the demand for the product is elastic with respect to price. Buyers are more likely to purchase products when their prices decrease and less likely to purchase them when their prices increase. In contrast, when demand for a product remains relatively stable and buyers are not sensitive to price changes, the demand is price inelastic ( Collado *et al.,* 2017).

The pricing strategy is an important aspect of marketing as it affects both revenue and consumer behavior. The pricing environment is evaluated from the viewpoint of the firm and its strategies, as well as from the consumer’s perspective, with consideration of external influences such as government policies and competition ( Kotler *et al.,* 2022b). Buyers may be individuals or firms who purchase the entire product or only parts of it, aiming to satisfy their needs or requirements ( Subramanian and Azad, 2021). To meet these requirements, customers use various parameters such as price and quality to determine their purchasing decisions.

Different pricing models are analyzed after considering these factors, and considerations are given to their effective implementation, balancing price levels, and their impact on consumer behavior ( Geng *et al.,* 2018). Despite efforts of financial experts to understand pricing and estimation methods, reliable pricing models remain elusive ( Chen *et al.,* 2016).

*Pricing strategy*

Pricing strategy is an important part of a company’s marketing mix as it directly affects the company’s profitability and its competitiveness in the market. Before choosing an appropriate pricing strategy for its product or service, a company must consider several factors such as customer demand, production costs, and competition ( Kotler *et al.,* 2022a).

Businesses can use a variety of pricing strategies, including cost-plus pricing, value-based pricing, and penetration pricing. Cost-plus pricing involves adding a price to the cost of producing a product to determine the selling price. On the other hand, value-based pricing focuses on the perceived value of a product in the eyes of customers rather than the cost of production. Penetration pricing is often used to enter a new market, where a company sets a low price to attract customers and gain market share ( Kotler *et al.,* 2022a).

The choice of pricing strategy depends on the company’s objectives, the target market, and the competitive environment. For example, a hotel company can use a dynamic pricing strategy to adjust room rates based on occupancy levels and demand fluctuations ( Xiang *et al.,* 2017). Alternatively, luxury hotels may adopt a premium pricing strategy, charging high prices to reflect the exclusivity and quality of their services ( Luo *et al.,* 2019).

In summary, pricing strategy plays a vital role in a company’s marketing efforts, and a well-designed pricing strategy can help a company achieve its sales and revenue targets. However, market conditions, customer behavior and competition should be considered when selecting an appropriate pricing strategy for a product or service.

*Economy pricing*

According to Papatla and Zhang (2021), economic pricing is a pricing strategy when a product or service is offered at a lower price than competitors. Companies often use this tactic to attract price-sensitive customers and increase their market share. The success of economy pricing depends on several factors such as the company’s cost structure, elasticity of demand, and competition. Additionally, research by Papatla and Zhang (2021) highlights that economy pricing has a positive impact on customer satisfaction and loyalty, which can lead to long-term profitability for the business. In the hotel industry, according to Dai and Zhang (2017), economy pricing is used to balance room rates and room demand for future dates. The pricing strategies are influenced by the principles of supply and demand, with lower prices when the demand is low and higher prices when the demand is high. Neubert (2017) also noted that for pre-orders further from the arrival date, the probability of return is generally greater and prices tend to increase as the arrival date approaches.

In general, economy pricing is a widely used pricing strategy in various industries, including food retail and hospitality. The success of this pricing strategy depends on several factors, such as elasticity of demand, competition, and cost structure, and can have a significant impact on customer satisfaction and loyalty.

### Customer retention management

Effective customer retention management has become essential for businesses, especially in the hospitality industry. According to Ascarza *et al.* (2018), if the main objective of hotels is to attract customers, retaining them has become equally important. Research by Del Rio Olivares *et al.* (2018) shows that a 5% increase in customer retention can lead to increase of profit of 20% to 90%.

Therefore, companies need to manage their customers throughout their lifecycle, from suspect to partner or defender, as explained from Han and Hwang (2018).

In the past, many hotels did not prioritize guest satisfaction, leading to high churn, known as the ‘broken barrel’ hypothesis. As Hanaysha (2018) pointed out, this approach leads to a constant influx of new customers and the loss of existing customers. However, reducing churn can significantly improve profits. For example, Razzaq *et al.* (2018) found that a 5% reduction in churn can increase profits by 20% to 100%.

Several factors influence guest retention in the hospitality industry. According to Kung and Zhong (2017), adaptability and consistency in the decision-making process can affect customer loyalty patterns. Lee and Fay (2017) also highlight the importance of adaptability in the decision-making process as it shapes hotel strategy and processes. Moreover, the passive behavior of hotel staff greatly affects the level of customer loyalty ( Luo *et al.,* 2018). Luo *et al.* (2018) found that the best hotel staff do not always fully understand the importance and consequences of customer retention, as they often view it as just another characteristic of the hotel. Therefore, customer retention should be integrated into organizational goals and practices, and employees should be trained accordingly ( Reen *et al.,* 2017).

Leadership should also provide employees with control checks and learning opportunities to motivate them to prioritize customer retention ( Subrahmanyam, 2018). A combination of various strategies, including promotional offers, is the best way to retain customers in the hospitality industry ( Ferdous Azam and Karim, 2017).

Guest satisfaction and loyalty are key to improving hotel guest retention. Highly satisfied customers tend to remain loyal and contribute significantly to a hotel’s revenue by repeat purchases and recommending the hotel to others ( Kang and Kim, 2017). Additionally, satisfied customers are less likely to switch to competitors and are easier and cheaper to maintain, requiring less training and marketing effort ( Subrahmanyam and Azad, 2019).

With changing demographics, economics and competitive factors, the cost of acquiring new guests is increasing, forcing hotels to focus on profitable guest retention and building lasting relationships with them. Therefore, hotels must prioritize guest satisfaction and value to ensure guest retention and loyalty.

## Methods

### Ethical statement

This study received retroactive ethical approval from the council of ethics of University “Aleksander Moisiu” as this was a low-risk study. Written informed consent was obtained from all participants prior to completing the survey.

### Study design

This study uses a quantitative strategy because it uses statistics as a time-saving tool and requires less effort and resources. Moreover, using this method is the best way to quantify and analyze the relationship between variables. In this study, an attempt was made to quantify the economy pricing strategy as an independent variable to determine the relationship with customer loyalty in selected international hotels in Albania.

### Participant selection

Data collection took place from June 2022 to January 2023 in the cities of Tirana, Durres, and Vlora, where a purposive sampling strategy was employed to select seven international hotels as participants. Inclusion criteria for hotel selection were based on their international status and availability to accommodate data collection. The sample size was determined using the number of rooms in each hotel to ensure representation and diversity. Randomization was achieved by assigning a unique identification number to each hotel and using a random number generator to select the participating hotels. The method of random sampling aimed to provide equal opportunities for all customers to contribute to the study.

The selected hotels included Rogner, Sheraton, and Hilton in Tirana; Adriatik, Tropikal, and Palace in Durres; and Vlora International Hotel in Vlora. These hotels were chosen based on their relevance and representation within the international hotel sector in Albania.

By employing a purposive sampling strategy, randomization, and sample saturation, this study ensured the inclusion of diverse international hotels in the sample, increasing the generalizability of the findings. The rationale behind these methodological choices was to obtain a comprehensive understanding of the relationship between economy pricing strategies and customer retention in the international hotel industry in Albania.

### Data collection

As mentioned in the previous section, the study examines the relationship between economy pricing strategies of international hotels and customer loyalty in Albania using quantitative techniques and questionnaires. A survey consists of several questions asked of respondents based on their opinions and past experiences at a particular hotel. The study used surveys based on various scientific sources. The questionnaire was adapted from reliable academic work published in reliable international publications modified from Njeru and Karega (2017) and Rao and Kartono (2009). To ensure the survey’s suitability for the study’s context, the modified version was piloted prior to the main data collection. The pilot study involved a small group of participants who completed the survey and provided feedback on its clarity, relevance, and comprehensibility. Based on the pilot study’s findings, necessary revisions were made to enhance the questionnaire’s overall quality and ensure its alignment with the research objectives. The participants completed the survey and provided feedback on the aspects such as clarify, relevance and comprehensibility of the questions. The pilot study served as an iterative process to refine and improve the questionnaire. This iterative process allowed for the identification and resolution of any potential issues or ambiguities in the survey instrument, ensuring its effectiveness in collecting the desired data.

The language used in the modified questionnaire was Albanian for Albanian citizens and English for the foreigners. By adapting the survey to the local language, it was possible to better understanding and improve response rates among the Albanian population. And the questionnaire in English language gave the better understanding for the non-Albanian participants ( Trebicka and Tartaraj, 2023).

The survey was distributed in English language and Albanian language, to ensure maximum participation and understanding among the respondents. Language translation was carried out by bilingual experts to maintain the accuracy and integrity of the survey items.

To distribute the questionnaires, a systematic approach was adopted. The hotels were personally visited and selected international hotels in Tirana, Durres, and Vlora and the questionnaire were distributed to eligible respondents. The hotel management and staff were informed about the research objectives and provided support in ensuring the questionnaires reached the target participants. The survey administration followed ethical guidelines, ensuring voluntary participation and maintaining respondent confidentiality.

By modifying and piloting the survey instrument, utilizing established academic sources, employing a suitable language for distribution, and adopting a systematic approach to questionnaire administration, this study aimed to gather reliable and relevant data on the relationship between economy pricing strategies and customer loyalty in international hotels in Albania.

Each question in the questionnaire was rated using a Likert scale. The scale goes from 1 (totally disagree) to 5 (totally agree).

The study attempted to collect personal information about respondents in the first half of the survey, such as their age, level of education, gender and marital status. The second part of the survey included questions on economy pricing strategies and customer retention. The data collected from survey questionnaires were compiled into a consolidated dataset to facilitate efficient data management and analysis. In order to protect the privacy and confidentiality of the respondents, steps were taken to anonymize the data. Personal identifying information, such as names, addresses, and contact details, were removed or replaced with unique identifiers to ensure that individual participants could not be identified. After this, for the closed-ended questions, coding was applied to transform the responses into categorical values for analysis. This coding process involved assigning specific codes or categories to different response options to facilitate data analysis and interpretation.

The collected data, whether in electronic or paper format, were entered into a spreadsheet for further analysis. During the data entry phase, careful attention was paid to minimizing errors by implementing double-entry techniques. The data were securely stored and managed throughout the study. Backup copies of the data were created to prevent data loss or corruption. To ensure the accuracy and reliability of the data, a process of data validation and verification was carried out. This involved cross-checking the entered data against the original survey responses to identify any discrepancies or errors. Data cleaning techniques were employed to address missing or inconsistent data.

### Data analysis

Several statistical tests were performed using IBM SPSS Statistics software (version 27.0) to examine the relationships and test the research hypotheses. The reliability of the survey questions was assessed using the built-in reliability analysis test in SPSS. This test calculates various reliability measures, such as Cronbach’s alpha coefficient, to evaluate the internal consistency of the questionnaire items. The reliability analysis provides information on the reliability and consistency of the questions used in the study.

Cosstab and chi-square analysis: To explore the relationship between variables, crosstabulation (crosstab) and chi-square tests were conducted. These tests allow for the examination of associations and dependencies between categorical variables. The crosstab analysis provides a contingency table, and the chi-square test determines whether there is a statistically significant association between the variables.

Model summary: In the multiple regression analysis, the model summary provides information on the overall goodness of fit of the regression model. It includes measures such as R-squared, adjusted R-squared, and the F-test statistic, which indicate the proportion of variance explained by the independent variables and the overall significance of the regression model.

ANOVA tests were performed to assess the significance of differences among group means. In the context of the study, ANOVA could be used to examine differences in customer loyalty based on different levels of the independent variable (economy pricing strategy). It helps determine whether these differences are statistically significant.

By utilizing these statistical tests in SPSS, the study aimed to evaluate the reliability of the questionnaire, explore associations and dependencies between variables using crosstab and chi-square analysis, examine the overall goodness of fit of the regression model through model summary, and assess the significance of differences among group means using ANOVA. These analyses contribute to the understanding of the research hypotheses and provide insights into the relationships between variables.

## Results

The study involved a total of 572 participants from various demographic backgrounds, including age, gender, education, and marital status. Reliability analysis was used to assess the reliability of the questions used in this study, while relationship analysis was used to determine the correlation between the independent and dependent variables. Economy pricing strategy was used as the independent variable, while customer loyalty was the dependent variable. A multiple regression analysis was then performed to measure each developed research hypothesis based on the research model.

### Analysis of participants’ demographics

This section includes an analysis of demographic data collected from participants who participated in the study, such as age, education level, marital status, and gender.

*Distribution of clients by gender*

**Figure 2.**
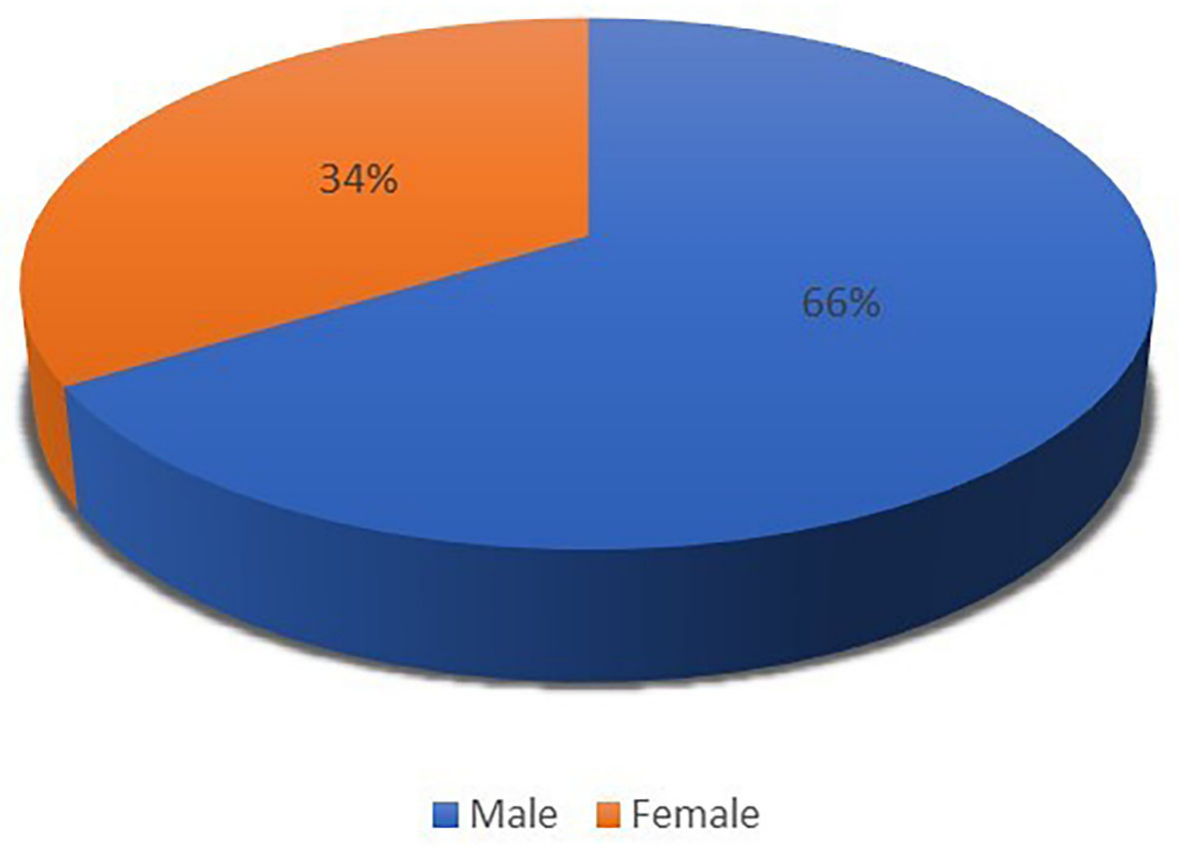
Distribution of customer gender (n=572).

Table 1 and Figure 2 illustrate the distribution by gender of clients participating in this study. Data was collected from seven international hotels in Albania. Of the 572 participants, there were 378 men and 194 women. As can be seen, most of the participants were men ( Trebicka and Tartaraj, 2023).

**Table 1.** Customer gender breakdown.

Customer gender	Frequency	Percentage
Male	378	66.08%
Female	194	33.92%
**Total**	**572**	**100.00%**

*Customer age*

**Figure 3.**
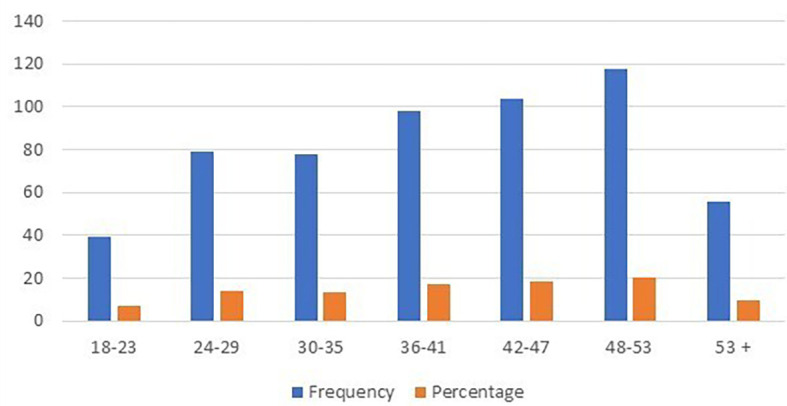
Customer’s age distribution (n=572).

From the data in Table 2 and Figure 3, we find that customer age has a significant effect. The study included participants staying at seven international hotels in Albania, covering a wide range of ages. Specifically, there are 39 people aged 18-23, 79 people aged 24-29, 78 people aged 30-35, 98 people aged 36-41, 104 people aged 42-47, 118 people between the ages of 48 and 53 and 56 participants aged 53 and over.

**Table 2.** Customer's age.

Age	Frequency	Percentage
18-23	39	6.82
24-29	79	13.81
30-35	78	13.64
36-41	98	17.13
42-47	104	18.18
48-53	118	20.63
53 +	56	9.79
Total	572	100.00

It is particularly important to note that the majority of respondents were between 48 and 53 years old. This finding suggests that this age group may be particularly important for hoteliers to meet the needs and preferences of their guests.

### Customers’ marital status

**Figure 4.**
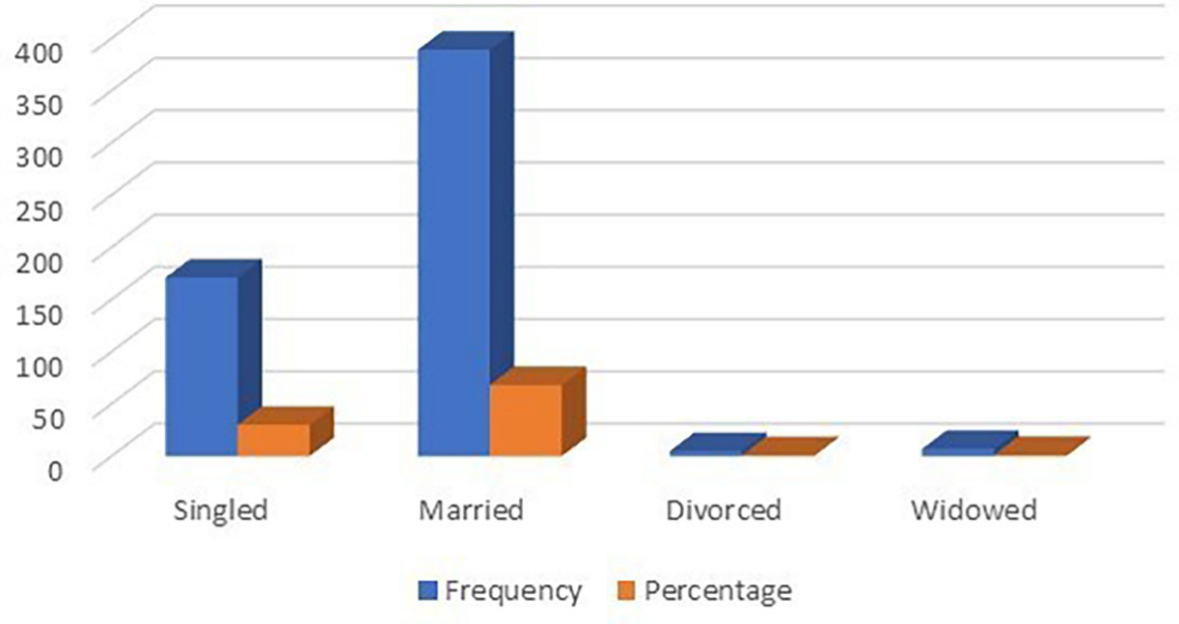
Customer marital status distribution (n=572).

From Table 3 and Figure 4, it can be seen that the visitor’s marital status plays a certain role in this study. A total of 389 married guests from seven international hotels in Albania participated in the study, 171 single guests from seven international hotels in Albania participated in the study, and 5 divorced guests from seven international hotels in Albania participated in the study. Married clients made up the majority of study participants.

**Table 3.** Marital status.

Customer marital status	Frequency	Percentage
Singled	171	29.90
Married	389	68.01
Divorced	5	0.87
Widowed	7	1.22
Total	572	100.00

### Customers’ education

**Figure 5.**
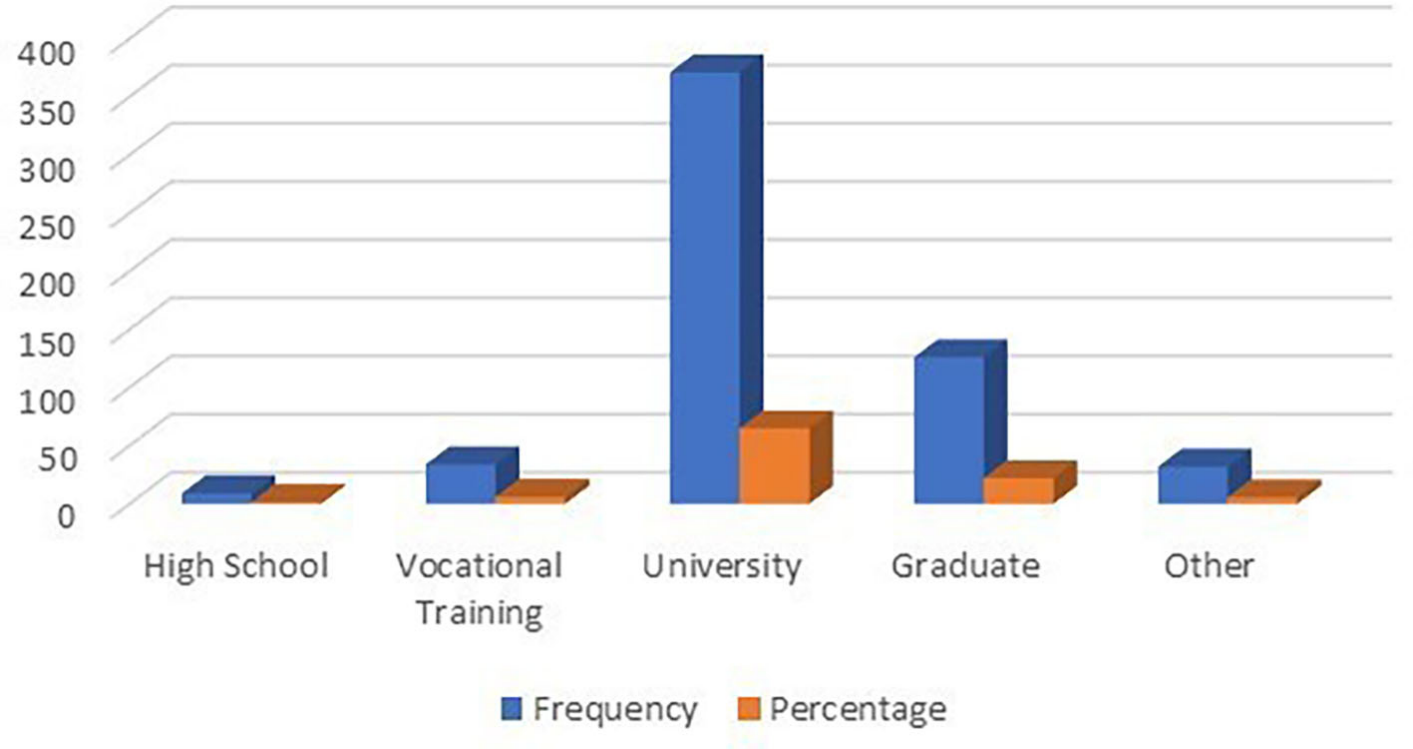
Customer’s education distribution (n=572).

According to Table 4 and Figure 5, it can be seen that customers’ educational backgrounds played a role in this research. A total of nine customers with high school diplomas from seven international hotels in Albania participated in the current research, 34 customers with vocational school certificates from the same hotels participated in the current study, and 371 customers with university degrees participated in the current study. Also, 126 guests with graduate degrees from seven international hotels in Albania participated, and 32 guests with certificates other than those listed participated. It may be concluded that the majority of participants were university graduates.

**Table 4.** Customer's education.

Customer education	Frequency	Percentage
High School	9	1.57
Vocational Training	34	5.94
University	371	64.86
Graduate	126	22.03
Other	32	5.59
Total	572	100.00

### Hypotheses testing

In this section, data obtained through questionnaires from seven different international hotels in Albania are analyzed. As stated in the conceptual framework, the study aims to quantify and assess the resulting research hypotheses. The test of each research hypothesis are done with the use of reliability analysis to assess the dependability of the independent and dependent variables, a relationship method to evaluate the relationship between the independent and dependent variables, and multiple regression analysis to assess the developed research hypothesis.

Based on Table 5, the reliability test used to examine the four questions related to economy pricing shows that the first question, asked whether the specific international hotel is setting prices low to attract more customers to stay at their hotels, had an Alpha value of.842. The second question asks if a particular international hotel offers continuous discounts to its customers, with an alpha value of 0.851, considered a reliable economy pricing element to measure the relationship with customer loyalty for the survey in international hotels in Albania. The third question related to economy pricing asks whether a particular international hotel employs a pricing strategy that allows it to compete with other international hotels to attract more customers. It has an alpha value of 0.846, indicating that it is a reliable measurement. The fourth question asks if an international hotel has lowered the price of rooms by offering fewer services, such as rooms without breakfast. It has an alpha value of 0.847, which is also considered a reliable economy pricing element to study the relationship with customer retention rates in international hotels in Albania.

**Table 5.** Reliability analysis of economy pricing strategy and customer retention.

Questions	Cronbach's Alpha
Q1. This hotel does not offer discount	.842
Q2: This hotel makes a continuous effort to offer competitive pricing in the market	.851
Q3: The additional activities offered by this hotel (such as food, activities etc.) are reasonably priced	.846
Q4: This hotel does not compromise on services and facilities despite offering lower prices	.847

Source: SPSS.

### The relationship between the independent and dependent variable

**Figure 6.**
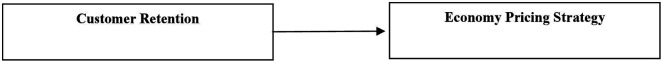
Relationship between customer retention and economy pricing strategy.

This section presents the findings of the relationship analysis conducted between the independent variable (economy pricing strategy) and the dependent variable (customer retention) ( Figure 6).

H _1_: There is a significant and positive relationship between economy pricing strategy and customer retention. Examining the relationship between the variables using a Crosstab as shown in Table 6 shows that out of 572 customers surveyed, 73 customers rated low concerning the relationship between economy pricing strategy and customer loyalty, 201 customers rated as fair concerning the relation between economy pricing strategy and customer retention at selected international hotels in Albania, and 298 customers rated high concerning the relation between economy pricing strategy and customer retention at selected international hotels in Albania. Based on the above results, we can conclude that the majority of customers rated high concerning the relationship between economy pricing strategy and customer retention at selected international hotels in Albania.

**Table 6.** Crosstab.

Crosstab					
count					
	Customer retention classes				
		Low	Fair	High	Total
Economic classes	Low	18	38	17	73
	Fair	3	62	136	201
	High	0	129	169	298
	Total	21	229	322	572

*Source:* IBM SPSS Statistics (version 27.0).

As the Table 7 shows, the chi-square statistic appears in the value column of the chi-square test table, just to the right of ‘Pearson chi-square’. The results show that the chi-square statistical value is 115.897. The p-values are in the same row of the Asymptotic Significance (2-sided) column (.000).

**Table 7.** Chi-square tests.

Chi-square tests			
	Value	df	Asymptotic Significance (2-sided)
Pearson-Chi Square	115.897	4	0.000
Likelihood Ration	86.258	4	0.000
Linear by Linear Association	71.5187	1	0.000
No. of valid cases	572		

*Source:* IBM SPSS Statistics (version 27.0).

This result is significant because the P value is less than 0.05. This demonstrates a significant relationship between economy pricing strategies and customer retention rates in selected international hotels in Albania.

### Multiple regression analysis

This section presents the results of multiple regression analysis to measure the research hypothesis developed according to the research model.

Model Summary Based on the model summary is shown in the Table 8, the value of Adjusted R Square for the regression analysis is.690. This suggests that 69% of the changes in the dependent variable can be explained by the changes of independent variables included in the model.

**Table 8.** Model summary.

Model summary				
Model 1	R	R square	Adjusted R Square	Std. The error in the estimate
1	0.829	0.695	0.690	2.515

*Source:* IBM SPSS Statistics (version 27.0).

### ANOVA

As presented in Table 9, the ANOVA analysis was conducted and it revealed that the F-value was 162.876, and the significance level was .000. Since the p-value is less than .05, it can be concluded that there is a statistically significant positive association between each pricing variable and customer retention at selected international hotels in Albania.

**Table 9.** ANOVA.

ANOVA						
	Model	Sum of Squares	df	Mean Square	F	Sig.
1	Regression	5901.626	6	983.604	162.867	.000
	Residual	2593.741	410	6.326		
	Total	8495.367	416			

### Coefficients

Multiple regression tests were performed between economy pricing strategy as an independent variable and customer loyalty as a dependent variable to test the research hypotheses previously developed for selected international hotels in Albania. As shown in Table 10, multiple regression analysis revealed a significant relationship between economy pricing strategies and customer retention. The B value is 0.238, the Beta value is 0.116, and the significance level is.000.

**Table 10.** Coefficients.

	Coefficients					
	Model	Unstandardized coefficients		Standardized coefficients		
		B	Std. Error	Beta	t.	Sig.
1	Economy	0.238	0.072	0.116	3.296	0.000

Source: SPSS.

This result suggests a positive relationship between economy pricing strategies and customer loyalty. The results support the research hypothesis that there is a positive and significant relationship between economy pricing strategies and customer loyalty in Albania.

## Discussion

Many external factors can affect customer retention in the hotel industry in Albania. Pricing is a critical step in building loyalty and retention of hotel guests. Effective pricing strategies have been developed to facilitate the development of international hotel services in a competitive environment. Therefore, this study aims to investigate the existing economy pricing strategies to determine the level of usage and its impact on customer retention in the international hotel sector in Albania. The results of this study will provide valuable information to scholars and researchers interested in conducting research in similar areas.

The research findings will be significant for the hotel industry in Albania as they will be able to use the insights to develop better pricing strategies to retain customers

The results of this study will guide how the hospitality industry in Albania can use pricing strategies as a retention strategy, identifying any gaps that may prevent retention and working towards improving customer retention. Business leaders will also have more insight into which retention strategies are being used well and which are not. The findings of this study will help business leaders take the right steps to maintain market share to improve their performance.

In this study, the independent variable is economic pricing and the dependent variable is customer retention.

According to the research hypothesis, there is a significant positive relationship between economy pricing strategies and customer retention. To know the relationship between the economy price of international hotels in Albania and the rate of customer retention, the Crosstab test was used.

The results are of great importance for the hotel sector in Albania, as they will be able to use this information to develop better pricing strategies to retain customers. Furthermore, the findings of the study are valuable for Albania’s tourism industry, which is an important sector of the country’s economy. The government can use the results to formulate policies that improve sector performance, leading to increase of economic growth.

Ultimately, the findings have important implications for the hotel industry and the Albanian tourism industry as a whole. The study will provide valuable insights into the effectiveness of economy pricing strategies in retaining customers in the Albanian international hotels. Based on the results, the hotel industry can develop better pricing strategies to improve customer retention and increase market share.

Despite the valuable insights gained from this study, several limitations should be acknowledged. Firstly, the research focused solely on international hotels in Albania, limiting the generalizability of the findings to other regions or types of accommodations. Secondly, the study relied on self-reported data collected through surveys, which may be subject to response bias or inaccuracies. Future research could employ a longitudinal approach to examine the long-term effects of pricing strategies on customer loyalty. Furthermore, incorporating qualitative methods or interviews could provide deeper insights into customers’ perceptions and experiences. Despite these limitations, the findings of this study contribute valuable knowledge to the field and offer a foundation for further investigations in pricing strategies and customer retention in the hotel industry.

## Conclusion

In conclusion, this study examines the impact of economy pricing strategies on customer retention in the international hospitality industry in Albania. The results show that a considerable number of customers perceive the relationship between economy pricing strategies and customer retention to be fair. Moreover, the statistical analysis revealed a significant positive relationship between economy pricing strategies and customer retention, which supports the research hypothesis.

These results provide valuable insights for business owners and hotel managers in Albania to use economy pricing strategies as a retention strategy and improve customer retention. Additionally, the study provides guidance on identifying gaps in pricing strategies that may prevent retention and working towards improving customer retention.

Furthermore, the study contributes to the existing literature on pricing strategies in the hotel industry and provides raw data for scholars and researchers interested in conducting research in similar areas. It should be noted that similar results have been found in previous studies using economy pricing in other industries.

In conclusion, the findings of the study have important implications for the international hotel industry in Albania, particularly in a highly competitive market environment. Using economy pricing strategies as a retention strategy can boost hotel services and improve their performance. The study also forms the basis for future research in the areas of pricing strategies and customer retention in the Albanian hotel industry.

## Data Availability

Figshare: Analyzing the Relationship Between Pricing Strategy and Customer Retention in Hotels: A study in Albania. https://doi.org/10.6084/m9.figshare.22814129.v1 ( Trebicka and Tartaraj, 2023). The project contains the following underlying data:
• Dataset.xlsx. (Anonymised data collected from questionnaires with coded responses). • Dataset.xlsx. (Anonymised data collected from questionnaires with written responses). Figshare: Analyzing the Relationship Between Pricing Strategy and Customer Retention in Hotels: A study in Albania. https://doi.org/10.6084/m9.figshare.22814129.v1 ( Trebicka and Tartaraj, 2023). This project contains the following extended data:
• Questionnaire English and albanian.docx. (Questionnaire in English and Albanian language). Figshare: SRQR checklist for Analyzing the Relationship Between Pricing Strategy and Customer Retention in Hotels: A study in Albania. https://doi.org/10.6084/m9.figshare.22814129.v1 ( Trebicka and Tartaraj, 2023). Data are available under the terms of the Creative Commons Attribution 4.0 International license (CC-BY 4.0).
