# Supplementary material for: Analyzing the relationship between pricing strategy and customer retention in hotels: A study in Albania
Source: F1000Res. 2023 Jun 16;12:690. [Version 1] doi: 10.12688/f1000research.132723.1 (PMC10521091; doi:10.12688/f1000research.132723.1)
Supplement: Peer review history

# Reviewer report: Reviewer response for version 1

**DOI:** 10.5256/f1000research.145667.r189945

**Published:** 2023-09-08

**Authors:** Visar Rustemi

**Affiliations:** 1 University of Prishtina, North Mitrovic, Kosovo (Serbia and Montenegro)

I recently had the opportunity to read the article titled "Analyzing: A study in Albania by Authors; Trebicka B, TARTARAJ A and Harizi A.

 I would like to share my positive impressions and highlight the valuable contributions made by this research.

- The article presents a comprehensive analysis of the relationship between pricing strategy and customer retention in the service sector – hotels mainly. The findings are significant and stresses the light on customer retention as a main and vital concept in the hospitality industry for building and sustaining long-term relationships.
- The authors' choice of methodology and research design is commendable. The study is well-structured and employs rigorous methods, ensuring the reliability and validity of the results. Statistical analysis, including crosstab tests, employed to explore the relationship between economy pricing strategies and customer retention enhance the reliability of the research design.
- This article stands out due to its originality and the unique perspective it brings to the field. The research addresses an important gap in the existing literature and provides valuable insights that contribute to the advancement of knowledge in existing a statistically significant positive relationship between economy pricing strategies and customer retention in international hotels in Albania. These findings underscore the importance of implementing effective pricing strategies to enhance customer loyalty and guide the development of improved strategies within the hotel industry. Effective pricing strategies play a crucial role in fostering customer loyalty.
- The article is exceptionally well-written, with clear and concise language that makes it accessible to a wide range of readers. The logical flow of ideas and the organization of the content further enhance the overall readability.
- The authors have done an excellent job of incorporating relevant and up-to-date references, which strengthens the credibility of their arguments and demonstrates a thorough understanding of the subject matter.

However, the study's limitations, primarily its focus on specific hotels in Albania, call for further research to validate the generalizability of these findings. The insights gained from this study inform policymakers and industry stakeholders in formulating strategies to enhance customer retention within the hospitality sector. I highly recommend this article to fellow researchers and scholars in the field. The research presented in this article is of great value and contributes significantly to the existing body of knowledge.

 Thank you to the authors for their valuable contribution and for sharing their research with the academic community. I look forward to future publications from this team.

Is the work clearly and accurately presented and does it cite the current literature?

Yes

If applicable, is the statistical analysis and its interpretation appropriate?

Yes

Are all the source data underlying the results available to ensure full reproducibility?

Yes

Is the study design appropriate and is the work technically sound?

Yes

Are the conclusions drawn adequately supported by the results?

Yes

Are sufficient details of methods and analysis provided to allow replication by others?

Yes

Reviewer Expertise:

NA

I confirm that I have read this submission and believe that I have an appropriate level of expertise to confirm that it is of an acceptable scientific standard.

# Reviewer report: Reviewer response for version 1

**DOI:** 10.5256/f1000research.145667.r189929

**Published:** 2023-07-26

**Authors:** Pratap Chandra Mandal

**Affiliations:** 1 Indian Institute of Management, Shillong, Shilllong, India

1. Only seven hotels from three regions of Albania are studied. Can the results of the study be extended to other locations of Albania?
2. How is the questionnaire developed?
3. It is obvious that lower prices will improve customer retention. So, what are the contributions of the authors?
4. The model in Figure 1 is too simplistic. Kindly develop an in-depth model for analysis. 
5. The study lacks in novelty.
6. The authors should highlight the research limitations, research contributions, research implications, and the avenues of future research.

Is the work clearly and accurately presented and does it cite the current literature?

Partly

If applicable, is the statistical analysis and its interpretation appropriate?

Partly

Are all the source data underlying the results available to ensure full reproducibility?

Partly

Is the study design appropriate and is the work technically sound?

No

Are the conclusions drawn adequately supported by the results?

Partly

Are sufficient details of methods and analysis provided to allow replication by others?

Partly

Reviewer Expertise:

Marketing, Marketing Intelligence, Marketing Communications, Services Marketing, Sustainable Marketing, Qualitative Methods in Marketing, Product Innovation and Management

I confirm that I have read this submission and believe that I have an appropriate level of expertise to state that I do not consider it to be of an acceptable scientific standard, for reasons outlined above.

# Reviewer report: Reviewer response for version 1

**DOI:** 10.5256/f1000research.145667.r183898

**Published:** 2023-07-26

**Authors:** Satya Subrahmanyam

**Affiliations:** 1 Business School, Catholic University, Jounieh, Mount Lebanon, Lebanon

Abstract: This review aims to comprehensively analyze the various strategies employed by hotels to enhance customer retention. The study investigates the effectiveness of these strategies using analytical tools while also incorporating a conceptual framework to provide a broader understanding. A slight mention of the theoretical framework that underpins the research is also included. The significance of this study lies in its contribution to the hospitality industry by shedding light on successful customer retention strategies. However, it should be noted that the literature review is somewhat concise and would benefit from the addition of more references. Furthermore, the authors' use of a systematic approach to distribute questionnaires to hotel customers raises some doubts, considering the transient nature of hostel customers who are typically travelers with specific purposes. Nevertheless, the data analysis approaches employed are deemed adequate and in line with the objectives of the study. However, there are resemblances to a previously published article titled "Economy Pricing Strategy and Customer Retention – An Analysis," which should be acknowledged and addressed.

 Introduction: The introduction provides a comprehensive overview of the importance of customer retention strategies in the hospitality industry, setting the stage for the study's objectives and scope. A conceptual framework is outlined to guide the research, emphasizing the factors that influence customer retention.

 Literature Review: While the literature review provides a brief and straightforward overview of existing studies on hotel customer retention strategies, it lacks depth and the inclusion of additional relevant references. To strengthen this section, the authors should consider incorporating more recent research and diverse perspectives.

 Theoretical Framework: The study briefly touches upon the theoretical framework that informs the research. A more comprehensive discussion of the underpinning theories, models, or concepts would enhance the clarity of the study's theoretical foundation.

 Methodology: The authors describe the systematic approach used to distribute questionnaires to hotel customers. However, the suitability of this approach is questionable, considering that hotel customers are typically transient and may not be inclined to participate in lengthy surveys due to their purposeful travel nature. Addressing this limitation and offering a justification for the chosen methodology would enhance the study's credibility.

 Data Analysis: The approaches used for data analysis are well-defined and appropriate for addressing the research objectives. By utilizing analytical tools, the study provides valuable insights into the effectiveness of various hotel customer retention strategies.

 Significance of the Study: While the abstract provides an overview of the study's scope, it lacks a clear statement on the significance of the research. The authors should emphasize how their findings can contribute to the hospitality industry and its implications for hotel management.

 Similarities with Previous Publication: It has come to light that the present study bears resemblances to a previously published article titled "Economy Pricing Strategy and Customer Retention – An Analysis." To maintain academic integrity, the authors must acknowledge this similarity and provide a clear distinction between their work and the referenced article.

 Conclusion: The conclusion provides a concise summary of the study's findings, emphasizing the importance of implementing effective customer retention strategies in the hospitality industry. However, it would benefit from a discussion of the study's limitations and avenues for future research.

 Overall, the review offers valuable insights into hotel customer retention strategies using analytical tools and a conceptual framework. To strengthen the manuscript, the authors should expand the literature review, provide a clear theoretical framework, highlight the significance of the study, and address concerns regarding the methodology and similarities with a previously published article. With these improvements, the study will be better positioned to contribute to the existing body of knowledge in the field of hospitality management.

Is the work clearly and accurately presented and does it cite the current literature?

Yes

If applicable, is the statistical analysis and its interpretation appropriate?

Yes

Are all the source data underlying the results available to ensure full reproducibility?

Yes

Is the study design appropriate and is the work technically sound?

Yes

Are the conclusions drawn adequately supported by the results?

Yes

Are sufficient details of methods and analysis provided to allow replication by others?

Yes

Reviewer Expertise:

H.R. Practices; Leadership; Management; Soft Skills & Human Resources Analytics.

I confirm that I have read this submission and believe that I have an appropriate level of expertise to confirm that it is of an acceptable scientific standard.
